# Quantifying raft proteins in neonatal mouse brain by 'tube-gel' protein digestion label-free shotgun proteomics

**DOI:** 10.1186/1477-5956-5-17

**Published:** 2007-09-24

**Authors:** Hongwei Yu, Bassam Wakim, Man Li, Brian Halligan, G Stephen Tint, Shailendra B Patel

**Affiliations:** 1 Division of Endocrinology, Metabolism and Nutrition, Medical College of Wisconsin, Milwaukee, WI 53226, USA; 2 Department of Biochemistry, Medical College of Wisconsin, Milwaukee, WI 53226, USA; 3 National Center for Proteomics Research, Biotechnology and Bioinformatics Center, Medical College of Wisconsin, Milwaukee, WI 53226, USA; 4 Research Service, Department of Veterans Affairs New Jersey Health Care System, East Orange, NJ 07018, USA, and Department of Medicine, UMDNJ-New Jersey Medical School, Newark, NJ 07103-2714, USA; 5 Department of Veterans Affairs, Clement J. Zablocki Medical Center, Milwaukee, WI 53295, USA; 6 Qilu Hospital, Shandong University, 44 West Wenhua Road, Jinan, 250012, P. R. China

## Abstract

**Background:**

The low concentration and highly hydrophobic nature of proteins in lipid raft samples present significant challenges for the sensitive and accurate proteomic analyses of lipid raft proteins. Elimination of highly enriched lipids and interfering substances from raft samples is generally required before mass spectrometric analyses can be performed, but these procedures often lead to excessive protein loss and increased sample variability. For accurate analyses of the raft proteome, simplified protocols are needed to avoid excessive sample handling and purification steps.

**Results:**

We have devised a simple protocol using a 'tube-gel' protein digestion that, when combined with mass spectrometry, can be used to obtain comprehensive and reproducible identification and quantitation of the lipid raft proteome prepared from neonatal mouse brain. Lipid rafts (detergent-resistant membranes using Triton X-100 extraction) prepared from neonatal mouse brain were directly incorporated into a polyacrylamide tube-gel matrix without prior protein separation. After in-gel digestion of proteins, nanospray LC-MS/MS was used to analyze the extracted peptides, and the resulting spectra were searched to identify the proteins present in the sample. Using the standard 'label-free' proteomics approach, the total number of MS/MS spectra for the identified proteins was used to provide a measure of relative protein abundances. This approach was successfully applied to lipid rafts prepared from neonatal mouse brain. A total of 216 proteins were identified: 127 proteins (58.8%) were predicted to be membrane proteins, or membrane-associated proteins and 175 proteins (~80%) showed less than a 2-fold variation in the relative abundance in replicate samples.

**Conclusion:**

The tube-gel protein digestion protocol coupled with nanospray LC-MS/MS (TubeGeLC-MS/MS) offers a simple and reproducible method for identifying and quantifying the changes of relative abundances in lipid raft proteins from neonatal mouse brain and could become a useful approach for studying lipid raft proteins from various tissues.

## Background

Lipid rafts are cholesterol- and sphingolipid-enriched specialized structures present in biological membranes [1-5] that can be isolated by various techniques. A common method for the isolation of the rafts is to prepare detergent-resistant membranes (DRMs) by extraction with the nonionic detergent Triton X-100 at cold temperature. Recent interest in lipid rafts arises from observations that some membrane proteins appear to partition preferentially into raft domains, and may require this environment for their biological activity [4,5]. Many previous studies have utilized two-dimensional gel electrophoresis (2DE) for proteomic profiling, but this method is limited by its lower sensitivity and it is often inefficient when analyzing raft proteins. Mass spectrometry (MS) has become a powerful tool for the analysis of complex protein mixtures. Proteomics profiling of either protein mixtures fractionated by 1DE or unfractionated protein mixtures by protease digestion and LC-MS/MS analysis has become increasingly popular. Peptides are identified by searching the resulting MS/MS spectra against protein sequence databases and protein presence is inferred from peptide presence. This general approach is referred to as 'top-down' or 'shotgun' proteomics. Several studies utilizing 1D gel filtration or in-solution protein digestion, combined with stable isotope labeling or label-free LC-MS/MS, have successfully profiled the protein composition and abundance in lipid rafts prepared from different biological sources [6-14]. However, quantitation of changes in the raft protein abundance under various experimental circumstances remains a major challenge. A number of technical factors are critical for analytical reliability, such as sample quality, reproducibility of the raft preparations, quality of the chromatography system, and the performance of the mass spectrophotometer. The most pressing problems for lipid raft proteomic investigations are those involving sample preparation and handling. Lipid raft samples prepared by different methods are composed of highly enriched lipids and low concentrations of hydrophobic proteins. Raft preparations also contain many non-proteinaceous substances including exogenous reagents, such as salts, buffers and detergents employed for sample preparation. These highly enriched lipids and non-protein components, or contaminants can often interfere with proteome analysis and their removal is a critical step before any proteome analysis can be performed. Although the low protein concentrations in raft samples do not present a limitation for analysis, methods used for removing lipids and other interfering substances from raft samples can lead to excessive protein loss. Thus, the process of lipid raft preparation suitable for mass spectrometry is a major factor in the variability of data obtained by these powerful proteomic techniques.

For accurate analyses of the raft proteome, a robust protocol avoids excessive purification steps, each of which lead to additional protein losses, is desirable. To avoid protein loss during sample preparation for mass spectrometry, a 'tube-gel' protein digestion protocol was adopted in which the lipid raft samples were directly incorporated into a polyacrylamide tube-gel without electrophoresis [15]. Detergents, lipids and other possible LC-MS/MS interfering materials in the raft samples are eliminated from the gel matrix while proteins are retained in the gel matrix. After the in-gel digestion of proteins, automated nanospray liquid chromatography tandem mass spectrometry (nanospray LC-MS/MS) is used to analyze the extracted peptides for protein identification. This protocol was used to analyze the protein profile of lipid rafts prepared from neonatal mouse brain. Neonatal mouse brain was chosen because there have been few proteomic studies of lipid rafts from neonatal brain [16-21]. Neonatal brain disorders are an important cause of mortality and morbidity contributing to the development of autism, cholesterol biosynthesis disorders, and a myriad of learning and developmental neurological and cognitive disabilities [22-27]. Developmental membrane defects have been postulated as one of the pathophysiological processes in these neonatal brain disorders. Additionally, the higher sterol content in brain tissue presents an additional challenge in preparing lipid raft samples for nanospray LC-MS/MS analysis.

Starting with limited amounts of frozen brain tissue, a total of 216 raft proteins were identified. Among the identified proteins, 127 (58.8%) were predicted to be plasma membrane (PM) or PM associated proteins including a number of authentic raft and/or GPI and lipid anchoring proteins, receptors, channel proteins, synaptic proteins, kinases, heterotrimeric G protein subunits, and some novel membrane proteins important for neurodevelopment. The major brain raft proteins, reported in previous investigations [8,18,19,28], were also identified as high abundance raft proteins in the present study. An advantage of this method is that it allows for raft proteins to be digested directly, dramatically reducing variations due to sample preparation prior to mass spectrometry. In this study, the standard 'label-free' proteomics approach in which total MS/MS spectral count is utilized to quantify the relative abundance of the identified proteins was used [29]. The results showed that the variations of relative abundance in ~80% of the identified proteins in replicate samples were less than 2-fold, suggesting that the method is highly reproducible. This approach offers a simple and reproducible protocol for identifying and quantifying changes in the relative abundance of the lipid raft proteins from neonatal mouse brain and could become a useful method for studying lipid raft proteins from various tissues.

## Results and Discussion

### Characterization of lipid rafts by sucrose density gradient ultracentrifugation

**Figure 1:**
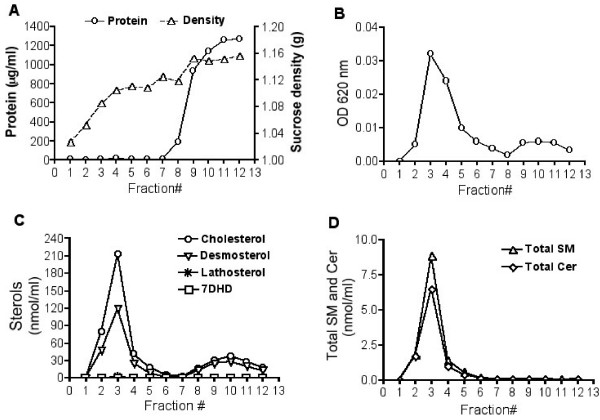
**Biochemical characterization of sucrose density gradient fractions of neonatal mouse brain**. Panel A shows the distributions of protein and sucrose density in membrane fractions from sucrose gradients of neonatal brain. Panel B shows the light-scattering properties of each fraction by absorbance at 620 nm. The buoyant low density fractions 2~4 showed the greatest light-scattering properties at 620 nm, consistent with a high content of lipids. Panel C shows the distribution of sterols (cholesterol, desmosterol, lathosterol, and 7-dehydrodesmosterol (7DHD)) in each fraction from sucrose gradients of neonatal mouse brain and Panel D shows total sphingomyelin (SM) and ceramide (Cer).

**Figure 2:**
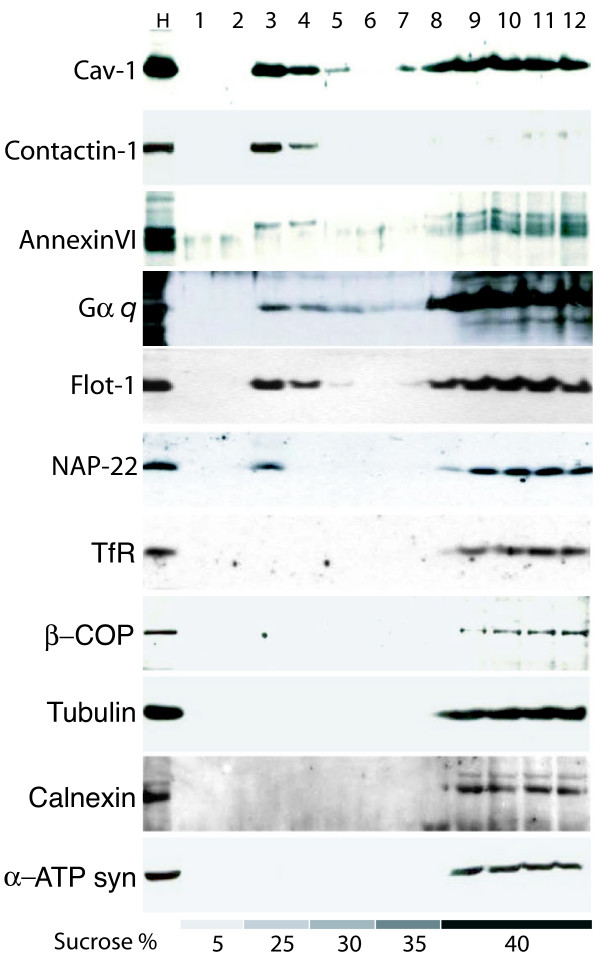
**Localization of known raft and non-raft marker proteins in sucrose gradients**. Post-nuclear homogenates (PNH) from neonatal brain tissues were extracted using 1% of Triton X-100 (TX) and fractionated in 5–40% discontinuous sucrose-density gradient as described in Methods. Twelve fractions of each 1.0 ml were collected from the top to bottom. Twenty μg of PNH protein (H) and equal 30 μl of each fraction of gradient were subjected to immunoblotting with antibodies against indicated proteins.

Biochemical isolation of lipid raft membranes by gradient ultracentrifugation, as well as their subsequent analysis, is a useful and simple method to determine if membrane components are located in raft microdomains. The distributions of total protein, sucrose density, and contents of sterols, sphingomyelin (SM) and ceramide (Cer), as well as the lighting-scattering properties at 620 nm, for each of the sucrose gradient fractions are summarized in Figure 1. Buoyant low density fractions 2–4 (DRMs/rafts) had the greatest light-scattering properties at 620 nm, consistent with a high content of lipids, but the non-raft fractions 8–11 had little or no absorbance. Conversely, most of the recovered proteins were present in the non-raft fractions and the total protein in the lipid raft fractions was too low to allow for accurate measurement by conventional methods. The lipid raft fractions 2~4 were highly enriched in sterols (a mixture composed of ~60% cholesterol, ~40% desmosterol, and trace amounts of other sterol precursors such as 7-dehydrodesmosterol and lathosterol), SM, and Cer compared to plasma membranes. Further characterization of the known raft and the non-raft marker proteins in the sucrose gradients was performed by immunoblotting (Figure 2). Known raft proteins, such as caveolin-1 (cav-1), flotillin-1 (flot-1), contactin-1 (Cntn-1), annexin -VI (Anx-VI, Anx6A), GTP-binding protein α*q *(Gα*q*), and NAP-22, were present in the low-density fractions (fractions 2~4). Various accepted non-raft markers, such as β-COP (a Golgi marker), transferrin receptor (TfR) (a non-raft membrane marker protein), α-tubulin (a cytoskeletal protein), calnexin (an ER resident membrane protein), and ATP synthase (a mitochondrial protein), were only present in the high-density fractions. Collectively, these results reflect the typical biochemical profiles of lipid rafts from brain tissue [8,30,31].

### Analysis of core raft proteome in neonatal mouse brain by TubeGeLC-MS/MS

The core protein composition of lipid rafts from neonatal mouse brain was determined by using a tube-gel protein digestion coupled with nanospray LC-MS/MS (TubeGeLC-MS/MS) analyses. The major benefit of this modification is that the raft proteins (usually in limiting quantities) are digested in a tube-gel matrix without fractionation and purification. Thus, sample losses are minimized compared to in-gel digestion based on SDS-PAGE. Moreover, inclusion of detergents (Triton X-100 and SDS. See Methods) can facilitate the effective solubilization and denaturation of hydrophobic lipid raft membrane proteins [32,33]. After proteins are incorporated into the tube-gel, the detergents, lipids and other interfering substances can be efficiently eliminated by extensive washing with acetonitrile prior to protein enzymatic digestion and subsequent nanospray LC-MS/MS analysis, without any significant loss of the proteins that are trapped in the gel matrix [15]. This tube-gel approach has been successfully employed for high throughout mass spectrometric analysis of membrane proteins [15].

**Figure 3:**
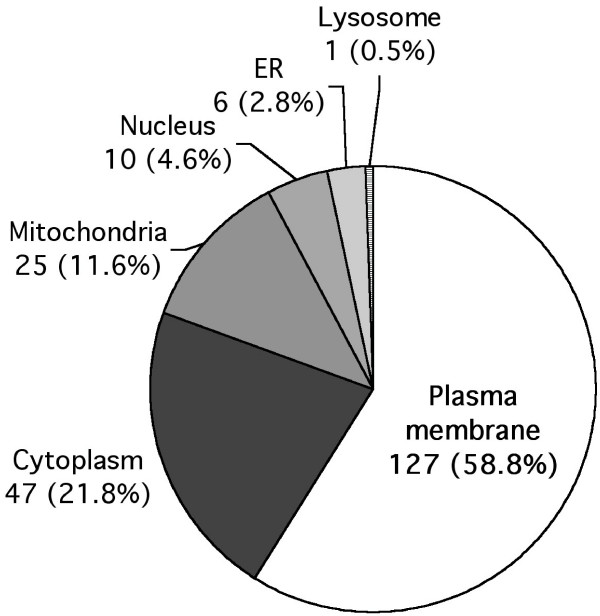
**Cellular localization of identified proteins in lipid rafts from neonatal mouse brain**. Cellular localization was annotated based on Gene Ontology (GO) terms and the PubMed literature database. The number of proteins and their percentage of the total identified proteins associated with each cellular location are indicated.

Two biological replicates (raft preparations from two neonatal brains), each with two MS technical replicates, were analyzed by TubeGeLC-MS/MS method. The peptides and corresponding proteins that were commonly identified in two biological samples (total 4 replicates) were considered as confident protein identifications. All identified proteins were then searched using UniProtKB/Swiss-Prot Release 52.3, TMHMM 2.0, and PubMed, to obtain information about their subcellular localization. The presence of predicted or verified transmembrane domains, glycosylphosphatidylinositol (GPI)-anchors and the lipid consensus sequences for myristoylation, pamitolyation, geranylgeranylation, farnesylation, and prenylation was used to classify proteins as either a membrane protein, or a membrane-associated protein [31]. Identified proteins were also analyzed by UniProtKB for the predicted presence of these motifs in order to provide an additional criterion for the evaluation. The overall experimental results for lipid raft core proteome of neonatal mouse brain thus characterized are shown in Figure 3. The complete lists of identified proteins, categorized as plasma membrane (PM) or PM-associated proteins and non-PM proteins, are shown in Tables 1 and 2, respectively.

**Table 1:** Plasma membrane associated proteins identified in the lipid rafts of neonatal mouse brain

**Acc. No**.	**Protein name**	**Pep**^*a*^	**% cov**^*b*^	**MW (KDa)**	**PTM**^*c*^	**Spectral count**^*d*^	**SD**^*e*^
P12960	Contactin-1 (Neural cell surface protein F3)	26	35.03	113.4	GPI	174.0	4.0
Q91XV3	Neuronal axonal membrane protein NAP-22	8	66.52	22.1	Lipid anchor	125.0	0.6
P59216	G-protein G(o), alpha subunit 1	7	25.28	40.1	Lipid anchor	101.0	2.0
P14824	Annexin A6	21	41.43	75.8		99.0	22.0
Q8BLK3	Limbic system-associated membrane protein	5	17.94	38.0	GPI	66.0	1.0
Q80Z24	Neuronal growth regulator 1 (Neurotractin)	4	14.99	37.9	GPI	56.5	2.5
Q61330	Contactin-2	13	20.69	113.2	GPI	44.5	5.5
P38401	G-protein G(i), alpha-1 subunit	5	19.03	40.4	Lipid anchor	42.5	10.5
O08917	Flotillin-1	8	29.04	47.5		40.0	0.0
Q8BFZ9	SPFH domain-containing protein 2	3	11.30	38.9		38.0	1.2
Q9Z2S9	Flotillin-2 (Reggie-1) (REG-1)	6	22.22	41.7		36.5	1.5
P13595	Neural cell adhesion molecule 1, 180 kDa isoform	6	9.43	119.4		32.5	10.5
P38402	G-protein G(i), alpha-2 subunit	2	8.78	40.5	Lipid anchor	30.0	4.0
Q9Z1G4	Vacuolar proton pump subunit 1	7	9.43	96.5		28.5	0.5
Q62188	Dihydropyrimidinase-related protein 3 (DRP-3)	7	20.39	61.9		21.5	0.5
P27600	G-protein alpha-12 subunit (G alpha-12)	2	8.49	44.0	Lipid anchor	20.5	5.5
O70443	G-protein G(z), alpha subunit (G(x) alpha chain)	4	17.85	40.9		19.5	1.5
Q99PJ0	Neurotrimin	3	11.95	38.0	GPI	17.0	1.0
Q9WTR5	Cadherin-13	4	9.26	78.3	GPI	17.0	2.0
P21278	G-protein alpha-11 subunit	3	9.78	42.0		15.0	0.6
P51150	Ras-related protein Rab-7	3	19.90	23.5	Lipid anchor	14.5	0.5
Q8VDN2	Sodium/potassium-transporting ATPase alpha-1 chain	5	8.61	113.0		13.5	0.5
P32736	Opioid-binding protein/cell adhesion molecule	3	13.08	38.1	GPI	13.0	2.0
P21279	G-protein G(q), alpha subunit	3	13.64	41.5	Lipid anchor	13.0	0.0
P01831	Thy-1 membrane glycoprotein precursor (Thy-1)	3	26.71	18.1	GPI	12.5	0.5
P59729	Ras and Rab interactor 3	2	3.14	107.3		12.0	2.0
Q6TMK6	G-protein G(I)/G(S)/G(T) beta subunit	2	8.81	37.4		12.0	4.0
P06837	Neuromodulin (Axonal membrane protein GAP-43	2	18.14	23.6	Lipid anchor	11.5	0.5
Q6PIE5	Sodium/potassium-transporting ATPase alpha-2 chain	3	10.71	112.2		11.0	1.0
P62821	Ras-related protein Rab-1A	2	16.26	22.7	Lipid anchor	11.0	1.0
P53994	Ras-related protein Rab-2A	3	20.38	23.5	Lipid anchor	10.5	0.5
P38403	G-protein G(k), alpha subunit	3	13.64	40.6	Lipid anchor	10.0	2.0
Q8BKV1	Glypican-2 precursor	2	5.88	63.3	GPI	10.0	2.0
Q68FD5	Clathrin heavy chain	5	4.06	191.6		10.0	3.0
Q91X78	SPFH domain-containing protein 1	6	24.48	38.9		9.5	2.5
P39688	Proto-oncogene tyrosine-protein kinase Fyn	2	3.57	59.9	Lipid anchor	9.5	1.5
Q61735	Integrin-associated protein (IAP)	2	4.64	33.1		9.5	0.5
P97792	Coxsackievirus and adenovirus receptor homolog	2	6.04	39.9	Lipid anchor	9.5	1.5
Q9WUC3	Lymphocyte antigen Ly-6H precursor	2	12.32	14.67	GPI	9.5	1.5
Q6PIC6	Sodium/potassium-transporting ATPase alpha-3 chain	3	4.45	111.7		9.0	1.0
Q8BMT4	Leucine-rich repeat-containing protein 33	2	2.75	77.1		9.0	1.0
P48036	Annexin A5	5	21.07	35.8		9.0	1.0
P17182	Alpha-enolase	3	11.57	47.1		9.0	0.6
O08532	L-type calcium channel subunit delta	2	2.75	124.6		8.5	2.5
P63044	Vesicle-associated membrane protein 2 (VAMP-2)	3	35.09	12.7		8.0	0.0
P54227	Stathmin (Phosphoprotein p19)	2	14.97	17.3		8.0	1.0
P61027	Ras-related protein Rab-10	3	16.58	22.5	Lipid anchor	8.0	1.0
Q9QZF2	Glypican-1 precursor	3	8.99	61.4	GPI	8.0	2.0
Q3U1F9	Transmembrane phosphoprotein Cbp	2	7.48	46.5	Lipid anchor	7.0	0.6
Q69Z26	Contactin-4	3	2.15	117.5	GPI	7.0	1.0
Q9R0N7	Synaptotagmin-7 (Synaptotagmin VII) (SytVII)	2	7.71	45.5		6.5	0.5
Q9Z0P4	Paralemmin	3	10.73	41.6	Lipid anchor	6.5	1.5
O35454	Chloride channel protein 6 (ClC-6)	4	6.56	97.0		6.5	1.5
P51863	Vacuolar ATP synthase subunit d	2	6.86	40.3		6.0	0.6
P62814	Vacuolar ATP synthase subunit B, brain isoform	3	8.04	56.6		6.0	1.0
Q9ER00	Syntaxin-12	2	11.72	31.19		6.0	2.0
Q7SIG6	Development and differentiation-enhancing factor 2	2	2.84	106.8		6.0	1.0
Q6PHN9	Ras-related protein Rab-35	2	5.50	23.0	Lipid anchor	5.5	0.5
P63213	G-protein G(I)/G(S)/G(O) gamma-2 subunit	2	8.78	37.4		5.5	0.5
P14094	Sodium/potassium-transporting ATPase beta-1 chain	2	8.25	35.2		5.0	2.0
Q6PCX7	Repulsive guidance molecule A	2	6.62	50.0	GPI	5.0	2.0
P70296	Phosphatidylethanolamine-binding protein (PEBP)	3	28.65	20.7		5.0	3.0
P59108	Copine-2 (Copine II)	3	6.76	61.0		5.0	1.7
Q9WV55	VAMP-associated protein A	2	4.98	27.3		4.5	0.5
P46096	Synaptotagmin-1	2	5.71	47.4	Lipid anchor	4.5	1.5
Q9D1G1	Ras-related protein Rab-1B	2	16.50	22.2	Lipid anchor	4.5	0.5
P07356	Annexin A2 (Annexin II)	3	13.95	38.5		4.5	1.5
Q99KR6	RING finger protein 34	2	4.53	42.0		4.0	1.0
Q91V41	Ras-related protein Rab-14	2	12.62	23.9	Lipid anchor	4.0	1.2
P10852	4F2 cell-surface antigen heavy chain	2	5.14	58.3		4.0	0.6
P35279	Ras-related protein Rab-6A (Rab-6)	2	5.34	23.6	Lipid anchor	3.5	1.5
Q9CQD1	Ras-related protein Rab-5A	2	10.28	23.6	Lipid anchor	3.5	0.5
O35963	Ras-related protein Rab-33B	2	4.82	25.8	Lipid anchor	3.5	0.5
Q8K386	Ras-related protein Rab-15	2	5.21	24.3	Lipid anchor	3.5	1.5
P60764	Ras-related C3 botulinum toxin substrate 3	2	7.33	21.4	Lipid anchor	3.5	0.5
Q9QXL2	Kinesin family member 21A	2	1.14	186.53		3.5	0.5
Q8R4A8	G-protein G(s), alpha subunit	3	13.64	45.7	Lipid anchor	3.5	1.5
Q9JMB8	Contactin-6	3	3.21	113.8	GPI	3.5	2.5
Q65CL1	Alpha-3 catenin (Alpha T-catenin)	1	1.79	99.8		3.5	1.5
Q60547	Synaptonemal complex protein 3	3	6.01	27.1		3.0	0.6
Q9DAS9	G-protein G(I)/G(S)/G(O) gamma-12 subunit	4	22.86	78.7	Lipid anchor	3.0	1.0
P97449	Aminopeptidase N (Membrane protein p161)	1	1.87	109.7		3.0	1.0
Q9JHS3	Late endosomal/lysosomal Mp1-interacting protein	2	14.52	13.48		3.0	1.0
P31324	Prkar2b	1	4.11	46.04		3.0	1.5
P61264	Syntaxin-1B2 (Syntaxin 1B)	2	8.01	33.3		2.5	1.5
P35278	Ras-related protein Rab-5C	2	6.51	23.4	Lipid anchor	2.5	0.5
P63011	Ras-related protein Rab-3A	2	8.68	25.0	Lipid anchor	2.5	0.5
P97855	Ras-GTPase-activating protein binding protein 1	1	3.02	51.8		2.5	0.5
Q9CYH2	Protein C10orf58 homolog	2	5.99	24.4		2.5	0.5
P11505	Plasma membrane calcium-transporting ATPase 1	1	1.43	138.7		2.5	0.5
P05480	Neuronal proto-oncogene tyrosine-protein kinase Src	2	5.38	60.6	Lipid anchor	2.5	0.5
Q07310	Neurexin-3-alpha	1	4.11	174.0		2.5	0.5
O35136	Neural cell adhesion molecule 2	1	1.56	93.2	GPI	2.5	1.5
O89051	Integral membrane protein 2B	2	6.04	30.3		2.5	0.5
O08842	GDNF family receptor alpha-2	1	3.68	51.6	GPI	2.5	0.5
O08545	Ephrin-A3 precursor	2	8.60	21.2	GPI	2.5	0.5
Q9R1T7	Inducible T-cell co-stimulator (CD278 antigen)	2	8.54	225.30		2.5	0.5
P60879	Synaptosomal-associated protein 25	2	6.83	23.3	Lipid anchor	2.0	1.0
P80236	Ras-related C3 botulinum toxin substrate 1	3	18.42	8.8		2.0	1.0
P68404	Protein kinase C beta type	1	2.38	76.9		2.0	0.6
Q04690	Neurofibromin	2	3.81	319.6		2.0	0.6
Q60437	Insulin receptor substrate p53	2	6.15	57.64		2.0	0.6
Q61411	GTPase HRas	2	12.77	21.3	Lipid anchor	2.0	0.6
P51655	Glypican-4 precursor (K-glypican)	2	4.86	62.6	GPI	2.0	1.0
P23818	Glutamate receptor 1 (GluR-1)	1	2.21	101.57		2.0	0.6
Q8VBX4	C-type lectin domain family 4 member K	1	4.24	37.6		2.0	0.6
Q8JZW4	Copine-5 (Copine V)	1	2.53	65.6		2.0	1.0
Q8BLR2	Copine-4 (Copine IV)	1	2.70	62.4		2.0	0.6
Q02013	Aquaporin-1	1	7.49	28.66		2.0	1.2
Q07076	Annexin A7	1	3.46	49.9		2.0	1.7
P97429	Annexin A4 (Annexin IV)	3	10.09	35.9		2.0	0.6
Q9DBE8	Alpha-1,3-mannosyltransferase ALG2	1	3.86	47.4		2.0	1.0
P84078	ADP-ribosylation factor 1	2	6.15	20.6		2.0	1.0
Q6QIY3	Sensory neuron sodium channel	1	3.09	220.6		2.0	0.6
P49817	Caveolin-1	1	7.91	20.54		2.0	0.6
P18708	Vesicle-fusing ATPase	2	2.69	82.54		1.5	0.5
O70439	Syntaxin-7	2	5.41	29.8		1.5	0.5
P16546	Spectrin alpha chain, brain	1	1.24	274.7		1.5	0.5
Q9QZB0	Regulator of G-protein signaling 17	1	6.22	24.3		1.5	0.5
Q9JIR4	Regulating synaptic membrane exocytosis protein 1	2	6.07	179.7		1.5	0.5
Q05909	Receptor-type tyrosine-protein phosphatase gamma	2	9.72	161.2		1.5	0.5
P46638	Ras-related protein Rab-11B	2	9.72	24.5	Lipid anchor	1.5	0.5
P05696	Protein kinase C alpha type(PKC-alpha)	1	2.09	76.8		1.5	0.5
O35764	Neuronal pentraxin receptor	1	2.84	52.37		1.5	0.5
Q8CGK7	G-protein G(olf), alpha subunit	6	20.74	44.3		1.5	0.5
P50153	G-protein G(I)/G(S)/G(O) gamma-4 subunit	2	9.35	84.1	Lipid anchor	1.5	0.5
Q99KJ8	Dynactin subunit 2	1	4.75	44.0		1.5	0.5

^*a*^Pep: peptide counts; ^*b *^% cov: protein coverage%; ^*c *^PTM: posttranslational lipid modification, GPI and lipid anchor: myristoylation, pamitolyation, geranylgeranylation, farnesylation, and prenylation; ^*d *^Spectral count: total MS/MS spectral counts. Number represents mean value of 4 replicates; ^*c *^standard deviation of spectral counts in 4 replicates.

**Table 2:** Non-PM proteins identified in the lipid rafts of neonatal mouse brain

**Acc. No**.	**Protein name**	**Pep**^*a*^	**% Cov**^*b*^	**MW (KDa)**	**Loc**.^*c*^	**Spectral count**^*d*^	**SD**^*e*^
P69893	Tubulin beta-1 chain	12	38.60	49.67	cyto	262.0	14.0
P68361	Tubulin alpha-1 chain	9	30.44	50.15	cyto	175.0	20.0
Q71FK5	Actin, cytoplasmic 1 (Beta-actin)	5	21.39	41.74	cyto	102.5	4.5
Q03265	ATP synthase alpha chain	10	26.63	59.75	mc	63.0	4.0
P56480	ATP synthase beta chain	9	20.83	56.30	mc	51.5	5.5
P04104	Keratin, type II cytoskeletal 1	3	5.59	65.1	cyto	37.0	4.0
P62629	Elongation factor 1-alpha 1	3	9.33	50.11	cyto	36.0	5.0
P19378	Heat shock cognate 71 kDa protein	4	10.39	70.8	cyto	33.0	1.0
Q922U2	Keratin, type II cytoskeletal 5	2	4.15	61.8	cyto	26.5	6.5
P14873	Microtubule-associated protein 1B (MAP 1B)	7	4.34	270.41	cyto	25.5	3.5
Q6IFZ6	Keratin, type II cytoskeletal 1b	2	4.20	61.4	cyto	25.5	3.5
P97427	Dihydropyrimidinase-related protein 1	4	8.93	62.17	cyto	25.0	0.0
Q62188	Dihydropyrimidinase-related protein 3	7	20.39	61.94	cyto	21.5	0.5
P14733	Lamin-B1	5	12.80	66.66	nuc	19.0	3.0
P68372	Tubulin beta-2C chain	2	6.76	49.83	cyto	18.5	1.5
Q6IG00	Keratin, type II cytoskeletal 4	2	1.68	57.7	cyto	18.0	1.0
Q60932	Voltage-dependent anion-selective channel protein 1	7	38.31	32.35	mc	16.5	3.5
P48962	ADP/ATP translocase 1	3	11.15	32.77	mc	15.0	2.0
Q922F4	Tubulin beta-6 chain	2	3.81	50.09	cyto	14.5	0.5
Q04447	Creatine kinase B-type	3	12.63	42.71	cyto	13.5	2.5
P67778	Prohibitin	4	21.03	29.82	mc	12.5	1.5
Q10758	Keratin, type II cytoskeletal 8	2	3.33	53.9	cyto	12.5	0.5
Q61696	Heat shock 70 kDa protein 1A	2	5.78	70.08	cyto	12.0	1.0
O08553	Dihydropyrimidinase-related protein 2	5	13.84	62.17	cyto	11.5	2.5
P46633	Heat shock protein HSP 90-alpha (HSP 86)	2	3.56	84.72	cyto	10.5	2.5
P63101	14-3-3 protein zeta/delta	2	12.30	27.77	cyto	10.5	0.5
Q60930	Voltage-dependent anion-selective channel protein 2	3	14.29	31.73	mc	10.0	0.0
Q9ERD7	Tubulin beta-3 chain	2	9.35	50.42	cyto	10.0	1.0
P50672	Cytochrome c oxidase subunit 2	2	11.50	25.82	mc	9.0	0.0
Q9DCT2	NADH-ubiquinone oxidoreductase 30 kDa subunit	2	6.35	34.00	mc	8.5	0.5
P18760	Cofilin-1 (Cofilin, non-muscle isoform)	2	15.24	18.43	nuc	8.5	1.5
P07823	78 kDa glucose-regulated protein	5	11.18	72.38	er	8.5	1.5
P11497	Acetyl-CoA carboxylase 1	1	4.24	37.62	cyto	8.0	1.0
Q60931	Voltage-dependent anion-selective channel protein 3	3	13.83	30.75	mc	7.5	0.5
P09445	Elongation factor 2	2	2.92	95.27	cyto	7.5	0.5
P62977	Ubiquitin	1	21.33	8.57	cyto	7.0	1.0
P19783	Cytochrome c oxidase subunit IV isoform 1	1	7.14	19.53	mc	7.0	2.0
P14152	Malate dehydrogenase	1	3.61	36.35	mc	6.5	0.5
P11499	Heat shock protein HSP 90-beta	2	3.88	83.20	cyto	6.5	0.5
P12787	Cytochrome c oxidase polypeptide Va	1	10.42	16.03	mc	6.5	0.5
P31253	Ubiquitin-activating enzyme E1 X	2	7.35	50.99	cyto	6.0	3.0
P35564	Calnexin	2	4.92	67.28	er	6.0	1.0
Q91VD9	NADH-ubiquinone oxidoreductase 75 kDa subunit	2	3.99	79.75	mc	5.5	2.5
P56135	ATP synthase f chain, mitochondrial	2	26.74	10.21	mc	5.5	1.5
P51881	ADP/ATP translocase 2	2	8.11	32.80	mc	5.5	0.5
Q8R429	SR Ca(2+)-ATPase 1	2	3.12	109.43	er	5.0	1.0
Q9DB20	ATP synthase O subunit	2	9.91	23.36	mc	5.0	1.0
Q8R429	Calcium pump 1 (SERCA1)	2	3.12	109.43	er	5.0	1.0
Q91V61	Sideroflexin-3	1	4.06	35.41	mc	4.5	0.5
P03995	Glial fibrillary acidic protein, astrocyte (GFAP)	1	2.56	49.92	cyto	4.5	1.5
Q9CQV8	14-3-3 protein beta/alpha	1	5.43	21.22	cyto	4.5	2.5
P68368	Tubulin alpha-4 chain	2	3.11	50.14	cyto	3.5	0.5
P62962	Profilin-1 (Profilin I)	2	21.74	14.83	cyto	3.5	0.5
Q8QZT1	Acetyl-CoA acetyltransferase	2	7.09	44.82	mc	3.5	0.5
P62962	Profilin-1	2	21.74	14.82	cyto	3.5	0.5
Q02053	Ubiquitin-activating enzyme E1 1	3	4.82	117.81	cyto	3.0	1.0
P42932	T-complex protein 1 subunit theta	3	6.59	59.43	cyto	3.0	2.0
O35129	Prohibitin-2	4	21.03	29.82	mc	3.0	1.0
P31324	Prkar2b	1	4.11	46.04	cyto	3.0	0.0
P20357	Microtubule-associated protein 2 (MAP 2)	2	1.20	198.98	cyto	3.0	0.0
P34926	Microtubule-associated protein 1A (MAP 1A)	2	2.36	299.53	cyto	3.0	0.0
P52480	Pyruvate kinase isozyme M2	4	12.48	57.76	mc	3.0	2.0
P63209	S-phase kinase-associated protein 1A	1	9.32	18.53	cyto	3.0	1.0
P60879	Synaptonemal complex protein 3	3	6.01	27.1	nuc	3.0	1.0
O88809	Neuronal migration protein doublecortin	1	3.56	40.61	cyto	2.5	0.5
P17156	Heat shock-related 70 kDa protein 2	2	3.01	69.74	cyto	2.5	1.5
Q9EQF6	Dihydropyrimidinase-related protein 5	1	3.20	61.52	cyto	2.5	0.5
Q8BH59	Calcium-binding mitochondrial carrier protein Aralar1	2	4.59	74.57	mc	2.5	0.5
P48670	Vimentin	1	4.25	51.85	cyto	2.5	1.5
P80315	T-complex protein 1 subunit delta	1	2.98	57.94	cyto	2.0	0.0
P11984	T-complex protein 1 subunit alpha A	1	4.14	60.34	cyto	2.0	0.0
Q9JKK8	Serine-protein kinase ATR	1	2.36	84.26	nuc	2.0	0.0
Q04899	Serine/threonine-protein kinase PCTAIRE-3	1	3.78	51.85	nuc	2.0	1.0
Q99PT1	Rho GDP-dissociation inhibitor 1 (Rho GDI 1)	1	7.88	23.41	er	2.0	0.0
Q61879	Myosin-10	1	3.09	49.59	cyto	2.0	0.0
P24638	Lysosomal acid phosphatase	1	2.13	48.51	lysosome	2.0	1.0
O70251	Elongation factor 1-beta	2	12.56	24.56	cyto	2.0	0.0
Q9CPQ8	ATP synthase g chain, mitochondrial	1	18.63	11.43	mc	2.0	0.0
O35627	Orphan nuclear receptor NR1I3	1	2.52	40.89	nuc	2.0	1.0
P53026	60S ribosomal protein L10a	1	6.98	24.78	nuc	2.0	1.0
P97524	Very-long-chain acyl-CoA synthetase	1	2.58	70.69	er	1.5	0.5
Q01853	Transitional endoplasmic reticulum ATPase	2	3.11	89.18	cyto	1.5	0.5
Q99JR1	Sideroflexin-1	1	5.63	35.52	mc	1.5	0.5
Q62627	PRKC apoptosis WT1 regulator protein	1	4.53	35.87	nuc	1.5	0.5
Q9DCS9	NADH-ubiquinone oxidoreductase PDSW subunit	1	10.92	20.89	mc	1.5	0.5
P08249	Malate dehydrogenase	2	9.79	35.60	mc	1.5	0.5
Q8BGU5	Cyclin fold protein 1	1	5.00	39.39	nuc	1.5	0.5
Q8CEE6	PAS-kinase (PASKIN)	1	1.16	151.27	cyto	1.5	0.5
P35980	60S ribosomal protein L18	1	6.99	21.5	nuc	1.5	0.5

^*a*^Pep: peptide counts; ^*b *^% cov: protein coverage%; ^*c *^Loc.: subcellular localization. mc. mitochondria, cyto. cytoplasm, er. endoplasmic reticulum; ^*e*^Spectral count: total MS/MS spectral counts. Number represents mean value of 4 replicates; ^*c *^standard deviation of spectral counts in 4 replicates.

The identified core proteome in the lipid rafts of neonatal mouse brain covered a wide range of sizes (8.0~319.6 kDa). Up to 216 non-redundant proteins were identified from 100 μl of a lipid raft fraction by TubeGeLC-MS/MS, 75% of these proteins were identified by at least two peptide matches and 25% of those identified were based upon a single peptide match. Although protein identifications based upon a single peptide match may be problematic, this does not necessarily imply a potential false identification [34]. For example, caveolin-1 (cav-1) was detected in raft samples from neonatal mouse brain by immunoblots, but was represented in each of the 4 replicates identified by mass spectrometry by a single peptide match. Using the Gene Ontology (GO) classifications and PubMed database searches, 127 (58.8%) of the proteins identified were PM or PM-associated proteins, with 18 (14.2%) having a GPI-anchoring site and 34 (26.8%) with other lipid-anchoring sites as described above. Many of the PM proteins identified were reported previously as being lipid raft proteins by conventional biochemical procedures. Typical raft marker proteins, such as caveolin-1, flotillin-1 and -2, Fyn and Src, were identified in the lipid raft preparations from neonatal mouse brain. Functional categories revealed that the identified PM proteins cover a broad range of neural functions involving neurodevelopment. Several proteins are known to function as part of the neurotransmitter release and re-uptake machinery; 3 syntaxin (Stx) proteins, Stx1A, Stx1B, Stx7; synaptosomal-associated protein 25 (Snap25); synaptotagmin (Syt) proteins, Syt1 and Sty7; vesicle-associated membrane proteins (Vamp), Vamp1 and Vamp2; regulating synaptic membrane exocytosis protein 1 (RIM1); and the glutamate receptor (GluR1) were all present in the raft fractions. Relatively large numbers of guanine nucleotide-binding protein (G protein) isoforms and Ras subfamily of GTPases were identified; G(s)α, G(i)α1, G(i)α2, G(o)α, G(k)α3, G(olf)α, G(q)α, G(z)α, Gα11, Gα12, G(I)/G(S)/G(T)β1, G(I)/G(S)/G(O)γ2, G(I)/G(S)/G(O)γ4, G(I)/G(S)/G(O)γ12 and Rab1A, Rab1B, Rab2A, Rab3A, Rab5A, Rab5C, Rab10, Rab11B, Rab14, Rab15, Rab33B, Rab35, p21Rac1, p21Rac3, Rab GDIα, RIN3, and G3BP. These proteins have been implicated in a variety of developmental processes in neonatal brain, including signal transduction, neurotransmitter release, and membrane trafficking [2]. Another important group of proteins in the neonatal brain-raft proteome comprises the cell adhesion/recognition molecules for cell-cell communication. Twenty-four such proteins were identified; contactin1 (Cntn1), Cntn2, Cntn4, and Cntn6, neurotrimin, Thy1, neurotractin (Kilon protein), Nap22, Gap43, paralemmin, desmocollin-2, neurexin-3α, Ncam2, Ncam180, dynactin, glypican (Gpc)1, Gpc 2, Gpc4, limbic system-associated membrane protein (LSAMP), transmembrane phosphoprotein Cbp, neurofibromin, opioid-binding cell adhesion molecule (Obcam), Alpha-3 catenin, and cadherin-13. These cell surface communication proteins are known to participate in the formation of neuronal networks in the brain during development, specifically axon growth, synapse formation, and fasciculation [35-38]. Several transporters and non-receptor type channel proteins were also identified; Na(+)/K(+) ATPase (ATP1A1, ATP1A2 and ATP1A3), small conductance calcium-activated potassium channel protein 3 (Kcnn3), plasma membrane calcium-transporting ATPase 1, Slc3a2, GDNF family receptor alpha-2, integrin-associated protein, integral membrane protein 2B, sensory neuron sodium channel, L-type calcium channel subunit delta, aquaporin-1, and chloride channel protein 6 (Clc-6). Calcium and phospholipid binding proteins cupine (Cnpe)2, Cnpe4, Cnpe591, annexin (Anx)2A, Anx4A, Anx5A, Anx6A, and Anx7A were also identified. A number of proteins of unknown function were also identified, such as receptor-type tyrosine-protein phosphatase gamma, protein C10orf58 homolog, Coxsackie's virus and adenovirus receptor homologs. As expected, since these raft proteins are from neonatal mouse brain, myelin proteins (present in adult brain tissue) such as myelin basic protein (MBP), myelin proteolipid protein (PLP), oligodendrocyte-myelin glycoprotein (Omg), and 2',3'-cyclic-nucleotide 3'-phosphodiesterase (CNPase), were not represented. Functional annotation and grouping of the major neonate-brain raft proteome will provide a basis for determining the potential targets of lipid raft disorganization in mouse models of neonatal brain disorders.

As reported in most raft proteomic studies [8,10,11,14,19,39-43], non-PM proteins were also found in the raft samples in the present study (Fig. 2 and Table 2). Eighty-nine of the 216 (41.2%) identified proteins from neonatal mouse brain rafts were predicted to be non-PM proteins by their GO terms. They are comprised of 47 cytoplasmic proteins including 20 cellular structural proteins (such as tubulins, actins, keratins, and microtubule-associated proteins), 25 mitochondrial proteins, 10 nuclear proteins, 6 ER proteins, and 1 lysosomal protein. Proteins from other subcellular compartments such as endosome and Golgi apparatus were poorly represented. The presence of subcellular membrane and cytoplasmic proteins in lipid raft fractions have been discussed in several proteomic studies [1,8,11,43-46]. One possibility is the contamination of non-plasma membrane proteins during gradient purification. The position of membrane particles in the density gradient ultracentrifugation is determined mainly by the ratio of its lipid and protein contents; different ratios of lipids to proteins for the various intracellular membrane particles could lead them to have different buoyant properties in density gradients. In this context, any method used for preparing cell membrane 'lipid rafts' is likely to generate a fraction containing membranes from a number of sub-cellular membranes, but not necessarily one enriched specifically in plasma membrane lipid rafts [8]. Certain subcellular proteins highly enriched in raft samples may be structurally involved and play critical roles in cell membrane lipid raft organization. For example, the cellular structural proteins such as tubulins, actins, keratins, and microtubular proteins, are highly enriched in lipid raft samples including brain-rafts as shown in this study and many other reports [7,8,13,28]. These cytoskeletal proteins not only contribute to the structural organization of cytoplasm but also play important roles in regulating the topography of the plasma membrane and trafficking and in modulating the localization of lipid raft proteins in eukaryotic cells [47,48]. Additionally, many proteins could have multiple cellular localizations regulated by multiple mechanisms. For example, cytoplasmic microtubule-associated proteins and 14-3-3 proteins, histones, and mitochondrial ATP synthases and voltage-dependent anion-selective channel 1 (VDAC1), have also been identified in cell plasma membranes [44,49-52]. Thus, enrichment of certain non-PM proteins in lipid rafts (DRMs) may represent a true observation of protein localization in different biological conditions and not necessarily be due to cross-contamination acquired during purification.

### Compilation of proteins into abundance lists

All proteins identified as PM protein or non-PM proteins in lipid rafts of neonatal mouse brain by TubeGeLC/MS/MS are compiled in Tables 1 and 2, respectively, and were sorted by their relative abundance calculated from the MS/MS spectral counts. Mass spectrometry of proteins and peptides is not quantitative, therefore, it is difficult to assess the abundance of a particular protein from the MS data *per se*. However, recent studies with label-free LC-MS/MS shotgun proteomics [29,53-57] revealed a relationship between protein abundance and sampling statistics, such as sequence coverage, peptide count, and spectral count. The use of sampling statistics is a promising method for measuring the relative protein abundance and detecting differentially expressed proteins. In general, the greater the amount of protein, the greater the MS signal intensity, number of spectral counts, sequenced peptides/sequence coverage, total ion current (TIC), and total Xcorr or scores that combine these values. Label-free proteomics has emerged as an alternative to stable isotope labeling for protein quantitation. The MS/MS spectral count, which compares the number of MS/MS spectra assigned to each protein, was selected for relative protein abundance in this study. Although this method has a tendency to overestimate the abundance of large proteins because they yield more peptides and therefore more spectral counts than the smaller proteins, the results indicate that this may not be a fundamental problem [29]. In the current study, contactin-1 (113.4 kDa) had a MS/MS spectral count of 174, but the sodium/potassium-transporting ATPase alpha-3 chain, a protein of almost identical size (111.7 kDa), had a spectral count of 9 (Table 1). It is reasonable to assume that the former protein is much more abundant than the latter. When plotting MS/MS spectral count *versus *protein size for all proteins identified (data not shown), both the maximum spectral count distribution was highest for proteins with a size distribution of 20~50 kDa. Therefore, the bias that may be potentially caused by size towards larger proteins may not be overly large, when using MS/MS spectral counts as a measure of abundance [29]. About 50% of proteins were identified with fewer than 5 total spectral counts, presumably due to their relatively low abundances. A total of 11 identified proteins with > 40 spectral counts were arbitrarily categorized as the most abundant proteins in the lipid rafts from neonatal mouse brain. These include contactin-1, NAP-22, Gα(o), annexin-A6, Lsamp, neurotractin, contactin-2, Gα(i), and flotillin-1, as well as intracellular structural and mitochondrial proteins such as tubulins, actins, and ATP synthases. Proteins with total spectral counts from 5 to 40 were arbitrarily categorized as medium abundance proteins. The relative abundances agree well with published data [7,18,19,30,39,40,42,43,45,49,58-71] and support our contention that the TubeGeLC-MS/MS approach provides a fair representation of the protein composition of the lipid rafts from neonatal mouse brain. The spectral count data for each identified protein provides proteome-wide semi-quantitative information on the relative abundance of lipid raft proteins.

### Comparison of protein identifications between GeLC-MS/MS and TubeGeLC-MS/MS

In-gel digestion can be efficiently employed after protein mixtures are resolved by SDS-PAGE or directly polymerized into a 'tube-gel' without electrophoresis [7,15]. Both of these in gel-based protein digestion protocols give clean LC-MS/MS baselines as interfering substances, such as detergents, salts and lipids, can be effectively removed during washing steps. To compare the GeLC-MS/MS versus the TubeGeLC-MS/MS, four separate experiments were conducted using 100 μl of sucrose-gradient isolated rafts that were subjected to a 1D SDS-PAGE combined with nanospray LC-MS/MS spectrometry (GeLC-MS/MS) modified by an established protocol [72], as described in Methods. The results for the peptides and corresponding proteins that were identified in a minimum of 2 of 4 independent experiments were used for comparative analyses. The comparison showed that about 200 proteins could also be identified by GeLC-MS/MS approach, with similar protein identifications, especially for high and medium abundance proteins, as compared to TubeGeLC-MS/MS (data not shown). However, the reproducibility of protein identified by GeLC-MS/MS was less than TubeGeLC-MS/MS (see below).

### Reproducibility of raft proteome characterization by TubeGeLC-MS/MS

To test the reproducibility of proteins identified, 3 raft samples from 3 separate neonatal brains prepared by identical methods at the same time were processed by both TubeGeLC-MS/MS and GeLC-MS/MS protocols, and the resulting protein identifications for within technique variations compared. There was a 68.8 ± 6.5% (SD) concordance in the proteins identified by TubeGeLC-MS/MS protocol among the 3 raft samples. As expected, the high abundance proteins showed a higher reproducibility of identification. The non-concordant proteins of ~30% may reflect some false identification because 55% of the non-concordant proteins had single or two peptide identifications. In addition, lipid raft isolations per se have a degree of variability. The results from the GeLC-MS/MS protocol yielded 45 ± 11% of concordance for within technique protein identifications.

**Figure 4:**
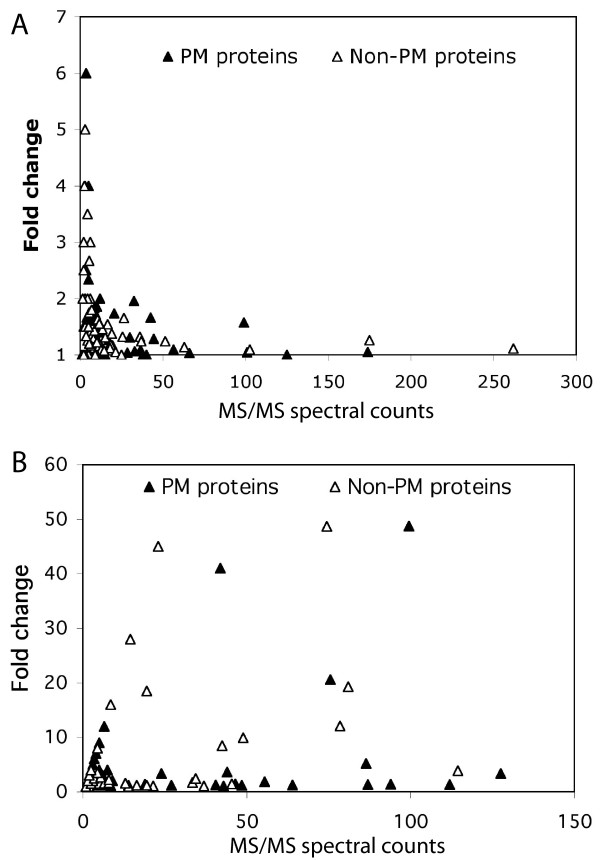
**Variations of quantification by spectral counting**. The variations in spectral counts for each protein were compared between the TubeGeLC/MS/MS and the GeLC/MS/MS protocols. The ratio of the spectral count per protein between two samples is presented as fold-change and plotted against the average of the spectral count of two samples. Panel A shows the results from the TubeGeLC/MS/MS method and panel B from the GeLC/MS/MS protocol.

A MS/MS spectral-count method was employed as a semi-quantitative measure for comparing proteins in different samples. Variability in protein abundance, calculated as MS/MS spectral counts, between the brain raft samples from two separate animals was evaluated and compared between the two approaches. The ratio of the spectral count per protein between these two samples was presented as fold-change and plotted against the average of the spectral count of the two samples. With the TubeGeLC-MS/MS method the fold-change was less than 2 for ~80% of the identified proteins; the higher the abundance, the lower of fold changes as shown in Figure 4A. However, greater variations for low abundance proteins were evident, indicating that the sensitivity of quantifying changes for low abundance proteins was generally lower. The fold-change results of the same samples analyzed by the GeLC/MS/MS protocol are shown in Figure 4B; greater variations were evident for both high and low abundant proteins. These results suggested that there was larger experimental variation associated with 1D gel protein separation and extraction from the gel slices prior protein digestion and mass spectrometry using the GeLC/MS/MS method. One of the explanations is that lipid associated proteins and other hydrophobic proteins may not fully enter the gel lanes in the GeLC/MS/MS protocol, causing variations in quantitative analyses. Employing the TubeGeLC-MS/MS approach, despite the experimental variation in isolating the lipid rafts, the protein composition from replicate samples was less variable, indicating that this simple change in sample handling results in more reproducible results.

## Conclusion

We have successfully combined a 'tube-gel' protein digestion protocol with nanospray LC-MS/MS analysis to carry out a high throughput proteomic mapping of lipid raft proteins isolated from neonatal mouse brain. Characterization of analytically difficult lipid raft proteins was simplified by this method. The MS/MS spectral count information from mass spectrometric analyses allowed for the label-free quantitation of relative protein abundances of more than 200 raft proteins from a single sample. The major advantage of this protocol is that the raft proteins are directly digested in a gel matrix without fractionation and purification, thus dramatically minimizing variation in protein yields due to losses during sample manipulation prior to mass spectrometry. With careful isolation of rafts, this protocol should allow for a reproducible quantitation of relative protein abundance in lipid rafts. This methodology should allow the investigation of the role of these specialized membranes under various biological conditions.

## Methods

### Reagents and antibodies

Sources for antibodies were as follows: caveolin-1 (Cav-1), contactin-1 (Cntn-1), annexin-VI (Anx VI, Anx6A), GTP-binding protein α*q *(Gα*q*), NAP-22, calnexin, α-tubulin from Santa Cruz Biotechnology, CA USA; flotillin-1 (flot-1): BD Transduction Laboratories, CA USA; mouse monoclonal antibody against β-COP: Sigma-Aldrich, MO USA; and mouse anti-human transferrin receptor antibody: Zymed Laboratories, CA USA. Trypsin Gold (MS grade) was obtained from Promega, WI, USA. All other reagents were from ThermoFisher Scientific, MA, USA.

### Preparation of raft-enriched detergent-resistant membranes from neonatal mouse brain

All animal experiments were performed with the approval of the Institutional Animal Care and Research Advisory Committee at the Clement Zablocki Veterans Medical Center. Neonatal mice (postnatal day 1, C57Bl/6J, Jackson Laboratories) were sacrificed by decapitation. Details of protocols used to prepare the raft-enriched detergent-resistant membranes have been described previously [30,73]. Briefly, frozen brains from neonatal mice, 50~60 mg of wet brain tissue, were homogenized in an ice-cold lysis buffer containing 5% glycerol in buffer A (50 mM Tris-HCl, pH 8.0, 10 mM MgCl_2_, 0.15 M NaCl, 20 mM NaF, 1 mM Na_3_VO_4_, 5 mM β-mercaptoethanol, 10 μg/ml aprotinin, 10 μg/ml leupeptin, 1 mM PMSF), using a tissue homogenizer (PRO Scientific Inc., Oxford, CT USA) by three pulses of 10 seconds each, followed by 20 strokes of a Dounce homogenizer, pestle A. Tissue debris and nuclei were removed by centrifugation at 1,000 *g *for 5 minutes and the pellet was re-extracted. The protein concentration of the post-nuclear homogenates (PNH) was measured using Protein Reagent (Bio-Rad, CA USA), adjusted to 2 mg/ml and 2 ml of the homogenates extracted with 1% Triton X-100 (TX) on ice for 30 min. The samples were mixed with an equal volume of ice-cold 80% (w/v) sucrose in buffer A, and then overlaid with 2.0 ml each of 35, 30, 25, and 5% (w/v) sucrose (all in buffer A). The sucrose gradient was centrifuged at 36,000 rpm in a Sorval 90 ultracentrifuge using a TH-641 rotor for 15 hr at 4°C. After ultracentrifugation, TX-resistant lipid rafts appeared as an insoluble white light-scattering band at the interface between the 5% and 25% sucrose layer. Twelve 1.0 ml fractions were collected from the top to bottom, with fractions 2–4 containing the rafts (density range of 1.055~1.115 g/ml). Non-raft fractions 8–11 were collected in the density range 1.130~1.180 g/ml.

### Tube-gel protein digestion

A Tube-Gel digestion method has been successfully used for high throughput analysis of membrane proteins and proven to be compatible with detergents in protein samples [15]. In these experiments, fraction 3 of the sucrose gradient was used as the lipid raft fraction. The raft fraction was directly incorporated into a polyacrylamide gel matrix as follows: 100 μl of the raft solution, 25 μl of acrylamide solution (40%, 29:1), 1.0 μl of 10% SDS, 0.5 μl of 10% ammonium persulfate, and 0.1 μl of TEMED were mixed in a 0.5 ml Eppendorf tube. The co-polymerization reaction was carried out for 30 min at room temperature. Post-polymerization, no liquid was extruded from the tube-gel, indicating that all of the materials were trapped in the gel matrix. The gel block was removed, cut into small pieces, and washed five times with 50% acetonitrile (v/v) in 25 mM ammonium bicarbonate for 15 min, using sonication and agitation. The gel pieces were dried using a SpeedVac, subjected to in-gel digestion using 100 μl of 10 ng/μl trypsin dissolved in 25 mM AMBIC and incubated at 37°C overnight. Peptides were then extracted from the gel using 500 μl of 0.1% formic acid in MS-grade water followed by 2 extractions with the same volume of 0.1% formic acid in 70% acetonitrile. Corresponding fractions were combined and dried using a SpeedVac. The dried samples were resuspended in 6 M guanidine-hydrochloride and 5 mM potassium phosphate, pH 6.0, purified using C-18 zip-tips from Millipore Corp., and subjected to nanospray LC-MS/MS analysis. This protocol is referred to as TubeGeLC-MS/MS.

Alternatively, lipid raft proteins were digested using an established protocol with some minor modification by 1-D electrophoresis coupled with nanospray LC-MS/MS (GeLC-MS/MS) [72]. Rather than conventional SDS-PAGE separation and multiple LC-MS/MS analyses, proteins in 100 μl of raft fractions, were first separated on 6% SDS-PAGE gels, long enough for the protein mixtures to penetrate the separation gel and then stained with silver. The stained areas of the gel containing the complex mixture of proteins were excised, digested with trypsin and applied to the nanospray LC-MS/MS to analyze raft proteome as described above.

### Nanospray LC-MS/MS spectrometry and data Analysis

Automated nanospray liquid chromatography tandem mass spectrometry (nanospray LC-MS/MS) was performed using an LTQ-LC/MS from ThermoFisher Scientific. Peptide mixtures were separated using a C18 reverse phase column (0.75-Å internal diameter at a flow rate of 1 μl/min) in line with the mass spectrometer. The mobile phases consisted of 0.1% formic acid containing 5% acetonitrile (A) and 0.1% formic acid in 95% acetonitrile (B), respectively. A 260-min linear gradient was typically used.

The MS data obtained were searched using the SEQUEST algorithm against the UniProt Rodent database v49.1. The search was limited only to tryptic peptides, and identifications were filtered from the search results using the Epitomize program [74]. Epitomize reads all the SEQUEST.out files in a directory, filters the files based on user-defined levels of Xcorr, and outputs the proteins identified. The Xcorr versus charge state filter used was set to Xcorr values of 1.8, 2.3 and 3.0 for charge states +1, +2 and +3, respectively. These filter values are similar to others previously reported for SEQUEST analyses [75]. Protein hits that passed the filter were annotated using the generic Gene Ontology (GO) slim. All proteins were identified by two or more peptides, and those identified with single peptide were included in the analysis if identified in two or more scans. Finally, the peptides listed were manually verified for correct identification by comparing the experimental spectra with the theoretical band ion spectra. Quantitative analyses were done using the open-source software program ZoomQuant, which provides a validation and a quantization platform for protein mass spectrometry [74,76].

### Biochemical analysis of lipids in gradient fractions

Sterol composition in each of the 12 fractions was quantitatively determined by gas chromatography/mass spectroscopy (GC/MS). An aliquot of ethanol containing the internal standard 5α-cholestane (25 μg) was added to each sample tube, and samples were hydrolyzed at 50°C in ethanol containing 1 M NaOH for 1 hour. Sterols were extracted in hexane (final volume 30 ml), dried under nitrogen, and derivatized with HMDS-TMCS. GC-MS analysis was performed using a Focus DSQ system (ThermoFisher Scientific). The trimethylsilyl-derived sterols were separated on a TR-35MS capillary column (35 m × 0.25 mm internal diameter × 0.25 μm film) with helium as the carrier gas at the rate of 1.8 ml/min. The temperature program was 150°C for 1 minute, followed by increases of 20°C/min up to 310°C, which was then held for 6 minutes. The injector was operated in the splitless mode at 250°C. Standard curves were generated by MS analysis of various amounts of each sterol. The contents of sphingomyelin (SM), ceramide (Cer) in each of the 12 fractions was quantitatively determined by LC/ESI/MS/MS on a Thermo Finnigan TSQ 7000 triple quadrupole mass spectrometer, operating in a Multiple Reaction Monitoring (MRM) positive ionization mode, as described previously [77].

### SDS/PAGE and immunoblots

A 30 μl aliquot of each fraction from the sucrose gradient was analyzed by SDS/PAGE on 10 or 12% (w/v) acrylamide gels. Separated proteins were transferred to nitrocellulose membranes for immunoblotting analyses. Membranes were blocked in 5% (w/v) non-fat milk in TBS-Tween [0.05% (w/v) Tween 20 in 10 mM Tris/100 mM NaCl, pH 7.5], and then incubated with the primary antibodies of choice. Membranes were subsequently incubated with HRP-conjugated second antibodies, and specific interactions were revealed using the ECL^® ^(Enhanced Chemiluminescence) detection system (Amersham, CA, USA).

## Competing interests

The author(s) declare that they have no competing interests.

## Authors' contributions

HY and BW conceived of the study and designed the experiments. HY, ML, and GST carried out the experiments. HY, BW, BH and SBP analyzed the data and prepared the manuscript. SBP supervised and coordinated the project and HY and SBP obtained funding for this project. HY and SBP wrote the manuscript, and all authors read and approved the final manuscript.

## References

[B1] Brown DA (2006). Lipid rafts, detergent-resistant membranes, and raft targeting signals. Physiology (Bethesda).

[B2] Allen JA, Halverson-Tamboli RA, Rasenick MM (2007). Lipid raft microdomains and neurotransmitter signalling. Nat Rev Neurosci.

[B3] Pike LJ (2004). Lipid rafts: heterogeneity on the high seas. Biochem J.

[B4] Simons K, Ehehalt R (2002). Cholesterol, lipid rafts, and disease. J Clin Invest.

[B5] Simons K, Vaz WL (2004). Model systems, lipid rafts, and cell membranes. Annu Rev Biophys Biomol Struct.

[B6] Blonder J, Hale ML, Lucas DA, Schaefer CF, Yu LR, Conrads TP, Issaq HJ, Stiles BG, Veenstra TD (2004). Proteomic analysis of detergent-resistant membrane rafts. Electrophoresis.

[B7] Martosella J, Zolotarjova N, Liu H, Moyer SC, Perkins PD, Boyes BE (2006). High recovery HPLC separation of lipid rafts for membrane proteome analysis. J Proteome Res.

[B8] Magee AI, Parmryd I (2003). Detergent-resistant membranes and the protein composition of lipid rafts. Genome Biol.

[B9] MacLellan DL, Steen H, Adam RM, Garlick M, Zurakowski D, Gygi SP, Freeman MR, Solomon KR (2005). A quantitative proteomic analysis of growth factor-induced compositional changes in lipid rafts of human smooth muscle cells. Proteomics.

[B10] Elortza F, Nuhse TS, Foster LJ, Stensballe A, Peck SC, Jensen ON (2003). Proteomic analysis of glycosylphosphatidylinositol-anchored membrane proteins. Mol Cell Proteomics.

[B11] Foster LJ, De Hoog CL, Mann M (2003). Unbiased quantitative proteomics of lipid rafts reveals high specificity for signaling factors. Proc Natl Acad Sci U S A.

[B12] Gupta N, Wollscheid B, Watts JD, Scheer B, Aebersold R, DeFranco AL (2006). Quantitative proteomic analysis of B cell lipid rafts reveals that ezrin regulates antigen receptor-mediated lipid raft dynamics. Nat Immunol.

[B13] Jia JY, Lamer S, Schumann M, Schmidt MR, Krause E, Haucke V (2006). Quantitative proteomics analysis of detergent-resistant membranes from chemical synapses: evidence for cholesterol as spatial organizer of synaptic vesicle cycling. Mol Cell Proteomics.

[B14] Sprenger RR, Horrevoets AJ (2007). Proteomic study of caveolae and rafts isolated from human endothelial cells. Methods Mol Biol.

[B15] Lu X, Zhu H (2005). Tube-gel digestion: a novel proteomic approach for high throughput analysis of membrane proteins. Mol Cell Proteomics.

[B16] Ishmael JE, Safic M, Amparan D, Vogel WK, Pham T, Marley K, Filtz TM, Maier CS (2007). Nonmuscle myosins II-B and Va are components of detergent-resistant membrane skeletons derived from mouse forebrain. Brain Res.

[B17] Kim KB, Lee JW, Lee CS, Kim BW, Choo HJ, Jung SY, Chi SG, Yoon YS, Yoon G, Ko YG (2006). Oxidation-reduction respiratory chains and ATP synthase complex are localized in detergent-resistant lipid rafts. Proteomics.

[B18] Kisby GE, Standley M, Park T, Olivas A, Fei S, Jacob T, Reddy A, Lu X, Pattee P, Nagalla SR (2006). Proteomic analysis of the genotoxicant methylazoxymethanol (MAM)-induced changes in the developing cerebellum. J Proteome Res.

[B19] Sheikh AM, Barrett C, Villamizar N, Alzate O, Miller S, Shelburne J, Lodge A, Lawson J, Jaggers J (2006). Proteomics of cerebral injury in a neonatal model of cardiopulmonary bypass with deep hypothermic circulatory arrest. J Thorac Cardiovasc Surg.

[B20] Spitzer AR, Chace D (2006). Mass spectrometry in neonatal medicine and clinical diagnosis--the [corrected] potential use of mass spectrometry in neonatal brain [corrected] monitoring. Clin Perinatol.

[B21] Yang ZJ, Appleby VJ, Coyle B, Chan WI, Tahmaseb M, Wigmore PM, Scotting PJ (2004). Novel strategy to study gene expression and function in developing cerebellar granule cells. J Neurosci Methods.

[B22] Kovacs WJ, Shackelford JE, Tape KN, Richards MJ, Faust PL, Fliesler SJ, Krisans SK (2004). Disturbed cholesterol homeostasis in a peroxisome-deficient PEX2 knockout mouse model. Mol Cell Biol.

[B23] Edison R, Muenke M (2003). The interplay of genetic and environmental factors in craniofacial morphogenesis: holoprosencephaly and the role of cholesterol. Congenit Anom (Kyoto).

[B24] Nissenkorn A, Michelson M, Ben-Zeev B, Lerman-Sagie T (2001). Inborn errors of metabolism: a cause of abnormal brain development. Neurology.

[B25] FitzPatrick DR, Keeling JW, Evans MJ, Kan AE, Bell JE, Porteous ME, Mills K, Winter RM, Clayton PT (1998). Clinical phenotype of desmosterolosis. Am J Med Genet.

[B26] Opitz JM, de la Cruz F (1994). Cholesterol metabolism in the RSH/Smith-Lemli-Opitz syndrome: summary of an NICHD conference. Am J Med Genet.

[B27] Powers JM, Tummons RC, Moser AB, Moser HW, Huff DS, Kelley RI (1987). Neuronal lipidosis and neuroaxonal dystrophy in cerebro-hepato-renal (Zellweger) syndrome. Acta Neuropathol (Berl).

[B28] Li N, Shaw AR, Zhang N, Mak A, Li L (2004). Lipid raft proteomics: analysis of in-solution digest of sodium dodecyl sulfate-solubilized lipid raft proteins by liquid chromatography-matrix-assisted laser desorption/ionization tandem mass spectrometry. Proteomics.

[B29] Old WM, Meyer-Arendt K, Aveline-Wolf L, Pierce KG, Mendoza A, Sevinsky JR, Resing KA, Ahn NG (2005). Comparison of label-free methods for quantifying human proteins by shotgun proteomics. Mol Cell Proteomics.

[B30] Mukherjee A, Arnaud L, Cooper JA (2003). Lipid-dependent recruitment of neuronal Src to lipid rafts in the brain. J Biol Chem.

[B31] Melkonian KA, Ostermeyer AG, Chen JZ, Roth MG, Brown DA (1999). Role of lipid modifications in targeting proteins to detergent-resistant membrane rafts. Many raft proteins are acylated, while few are prenylated. J Biol Chem.

[B32] Santoni V, Molloy M, Rabilloud T (2000). Membrane proteins and proteomics: un amour impossible?. Electrophoresis.

[B33] Blonder J, Chan KC, Issaq HJ, Veenstra TD (2006). Identification of membrane proteins from mammalian cell/tissue using methanol-facilitated solubilization and tryptic digestion coupled with 2D-LC-MS/MS. Nat Protoc.

[B34] States DJ, Omenn GS, Blackwell TW, Fermin D, Eng J, Speicher DW, Hanash SM (2006). Challenges in deriving high-confidence protein identifications from data gathered by a HUPO plasma proteome collaborative study. Nat Biotechnol.

[B35] Elmariah SB, Hughes EG, Oh EJ, Balice-Gordon RJ (2005). Neurotrophin signaling among neurons and glia during formation of tripartite synapses. Neuron Glia Biol.

[B36] Foty RA, Steinberg MS (2004). Cadherin-mediated cell-cell adhesion and tissue segregation in relation to malignancy. Int J Dev Biol.

[B37] Scheiffele P (2003). Cell-cell signaling during synapse formation in the CNS. Annu Rev Neurosci.

[B38] Fetissov SO, Bergstrom U, Johansen JE, Hokfelt T, Schalling M, Ranscht B (2005). Alterations of arcuate nucleus neuropeptidergic development in contactin-deficient mice: comparison with anorexia and food-deprived mice. Eur J Neurosci.

[B39] Yang JW, Rodrigo R, Felipo V, Lubec G (2005). Proteome analysis of primary neurons and astrocytes from rat cerebellum. J Proteome Res.

[B40] Dremina ES, Sharov VS, Schoneich C (2005). Protein tyrosine nitration in rat brain is associated with raft proteins, flotillin-1 and alpha-tubulin: effect of biological aging. J Neurochem.

[B41] Head BP, Patel HH, Roth DM, Murray F, Swaney JS, Niesman IR, Farquhar MG, Insel PA (2006). Microtubules and actin microfilaments regulate lipid raft/caveolae localization of adenylyl cyclase signaling components. J Biol Chem.

[B42] Chen S, Bawa D, Besshoh S, Gurd JW, Brown IR (2005). Association of heat shock proteins and neuronal membrane components with lipid rafts from the rat brain. J Neurosci Res.

[B43] Say YH, Hooper NM (2007). Contamination of nuclear fractions with plasma membrane lipid rafts. Proteomics.

[B44] Bae TJ, Kim MS, Kim JW, Kim BW, Choo HJ, Lee JW, Kim KB, Lee CS, Kim JH, Chang SY, Kang CY, Lee SW, Ko YG (2004). Lipid raft proteome reveals ATP synthase complex in the cell surface. Proteomics.

[B45] Igbavboa U, Eckert GP, Malo TM, Studniski AE, Johnson LN, Yamamoto N, Kobayashi M, Fujita SC, Appel TR, Muller WE, Wood WG, Yanagisawa K (2005). Murine synaptosomal lipid raft protein and lipid composition are altered by expression of human apoE 3 and 4 and by increasing age. J Neurol Sci.

[B46] Thouvenot E, Lafon-Cazal M, Demettre E, Jouin P, Bockaert J, Marin P (2006). The proteomic analysis of mouse choroid plexus secretome reveals a high protein secretion capacity of choroidal epithelial cells. Proteomics.

[B47] Pelkmans L, Puntener D, Helenius A (2002). Local actin polymerization and dynamin recruitment in SV40-induced internalization of caveolae. Science.

[B48] Chang L, Goldman RD (2004). Intermediate filaments mediate cytoskeletal crosstalk. Nat Rev Mol Cell Biol.

[B49] Franzen R, Tanner SL, Dashiell SM, Rottkamp CA, Hammer JA, Quarles RH (2001). Microtubule-associated protein 1B: a neuronal binding partner for myelin-associated glycoprotein. J Cell Biol.

[B50] Fu H, Subramanian RR, Masters SC (2000). 14-3-3 proteins: structure, function, and regulation. Annu Rev Pharmacol Toxicol.

[B51] Watson K, Edwards RJ, Shaunak S, Parmelee DC, Sarraf C, Gooderham NJ, Davies DS (1995). Extra-nuclear location of histones in activated human peripheral blood lymphocytes and cultured T-cells. Biochem Pharmacol.

[B52] Lawen A, Ly JD, Lane DJ, Zarschler K, Messina A, De Pinto V (2005). Voltage-dependent anion-selective channel 1 (VDAC1)--a mitochondrial protein, rediscovered as a novel enzyme in the plasma membrane. Int J Biochem Cell Biol.

[B53] Andreev VP, Li L, Cao L, Gu Y, Rejtar T, Wu SL, Karger BL (2007). A New Algorithm Using Cross-Assignment for Label-Free Quantitation with LC-LTQ-FT MS. J Proteome Res.

[B54] Wienkoop S, Larrainzar E, Niemann M, Gonzalez EM, Lehmann U, Weckwerth W (2006). Stable isotope-free quantitative shotgun proteomics combined with sample pattern recognition for rapid diagnostics. J Sep Sci.

[B55] Le Bihan T, Goh T, Salter AM, Bukhman YV, Dharsee M, Ewing R, Wisniewski JR, Stewart (2006). Differential analysis of membrane proteins in mouse fore- and hindbrain using a label-free approach. J Proteome Res.

[B56] Wang G, Wu WW, Zeng W, Chou CL, Shen RF (2006). Label-free protein quantification using LC-coupled ion trap or FT mass spectrometry: Reproducibility, linearity, and application with complex proteomes. J Proteome Res.

[B57] Ru QC, Zhu LA, Silberman J, Shriver CD (2006). Label-free semiquantitative peptide feature profiling of human breast cancer and breast disease sera via two-dimensional liquid chromatography-mass spectrometry. Mol Cell Proteomics.

[B58] Cui XY, Hu QD, Tekaya M, Shimoda Y, Ang BT, Nie DY, Sun L, Hu WP, Karsak M, Duka T, Takeda Y, Ou LY, Dawe GS, Yu FG, Ahmed S, Jin LH, Schachner M, Watanabe K, Arsenijevic Y, Xiao ZC (2004). NB-3/Notch1 pathway via Deltex1 promotes neural progenitor cell differentiation into oligodendrocytes. J Biol Chem.

[B59] Brady ST, Lasek RJ (1981). Nerve-specific enolase and creatine phosphokinase in axonal transport: soluble proteins and the axoplasmic matrix. Cell.

[B60] Deininger SO, Rajendran L, Lottspeich F, Przybylski M, Illges H, Stuermer CA, Reuter A (2003). Identification of teleost Thy-1 and association with the microdomain/lipid raft reggie proteins in regenerating CNS axons. Mol Cell Neurosci.

[B61] Hu QD, Ang BT, Karsak M, Hu WP, Cui XY, Duka T, Takeda Y, Chia W, Sankar N, Ng YK, Ling EA, Maciag T, Small D, Trifonova R, Kopan R, Okano H, Nakafuku M, Chiba S, Hirai H, Aster JC, Schachner M, Pallen CJ, Watanabe K, Xiao ZC (2003). F3/contactin acts as a functional ligand for Notch during oligodendrocyte maturation. Cell.

[B62] Guirland C, Suzuki S, Kojima M, Lu B, Zheng JQ (2004). Lipid rafts mediate chemotropic guidance of nerve growth cones. Neuron.

[B63] Kashihara M, Miyata S, Kumanogoh H, Funatsu N, Matsunaga W, Kiyohara T, Sokawa Y, Maekawa S (2000). Changes in the localization of NAP-22, a calmodulin binding membrane protein, during the development of neuronal polarity. Neurosci Res.

[B64] Leshchyns'ka I, Sytnyk V, Morrow JS, Schachner M (2003). Neural cell adhesion molecule (NCAM) association with PKCbeta2 via betaI spectrin is implicated in NCAM-mediated neurite outgrowth. J Cell Biol.

[B65] Maekawa S, Morii H, Kumanogoh H, Sano M, Naruse Y, Sokawa Y, Mori N (2001). Localization of neuronal growth-associated, microtubule-destabilizing factor SCG10 in brain-derived raft membrane microdomains. J Biochem (Tokyo).

[B66] Miyata S, Funatsu N, Matsunaga W, Kiyohara T, Sokawa Y, Maekawa S (2000). Expression of the IgLON cell adhesion molecules Kilon and OBCAM in hypothalamic magnocellular neurons. J Comp Neurol.

[B67] Roussel G, Nussbaum F, Schoentgen F, Jolles P, Nussbaum JL (1988). Immunological investigation of a 21-kilodalton cytosolic basic protein in rat brain. Dev Neurosci.

[B68] Schaeren-Wiemers N, Bonnet A, Erb M, Erne B, Bartsch U, Kern F, Mantei N, Sherman D, Suter U (2004). The raft-associated protein MAL is required for maintenance of proper axon--glia interactions in the central nervous system. J Cell Biol.

[B69] Schlicht K, Buttner A, Siedler F, Scheffer B, Zill P, Eisenmenger W, Ackenheil M, Bondy B (2007). Comparative proteomic analysis with postmortem prefrontal cortex tissues of suicide victims versus controls. J Psychiatr Res.

[B70] Yanagisawa M, Nakamura K, Taga T (2004). Roles of lipid rafts in integrin-dependent adhesion and gp130 signalling pathway in mouse embryonic neural precursor cells. Genes Cells.

[B71] Yang JW, Suder P, Silberring J, Lubec G (2005). Proteome analysis of mouse primary astrocytes. Neurochem Int.

[B72] Schirle M, Heurtier MA, Kuster B (2003). Profiling core proteomes of human cell lines by one-dimensional PAGE and liquid chromatography-tandem mass spectrometry. Mol Cell Proteomics.

[B73] Yu H, Li M, Tint GS, Chen J, Xu G, Patel SB (2007). Selective reconstitution of liver cholesterol biosynthesis promotes lung maturation but does not prevent neonatal lethality in Dhcr7 null mice. BMC Dev Biol.

[B74] Hicks WA, Halligan BD, Slyper RY, Twigger SN, Greene AS, Olivier M (2005). Simultaneous quantification and identification using 18O labeling with an ion trap mass spectrometer and the analysis software application "ZoomQuant". J Am Soc Mass Spectrom.

[B75] Mirza SP, Halligan BD, Greene AS, Olivier M (2007). An improved method for the analysis of membrane proteins by mass spectrometry. Physiol Genomics.

[B76] Halligan BD, Slyper RY, Twigger SN, Hicks W, Olivier M, Greene AS (2005). ZoomQuant: an application for the quantitation of stable isotope labeled peptides. J Am Soc Mass Spectrom.

[B77] Bielawski J, Szulc ZM, Hannun YA, Bielawska A (2006). Simultaneous quantitative analysis of bioactive sphingolipids by high-performance liquid chromatography-tandem mass spectrometry. Methods.

